# Inactivation Activities of Ozonated Water, Slightly Acidic Electrolyzed Water and Ethanol against SARS-CoV-2

**DOI:** 10.3390/molecules26185465

**Published:** 2021-09-08

**Authors:** Yohei Takeda, Dulamjav Jamsransuren, Yoshimasa Makita, Akihiro Kaneko, Sachiko Matsuda, Haruko Ogawa, Hourei Oh

**Affiliations:** 1 Research Center for Global Agromedicine, Obihiro University of Agriculture and Veterinary Medicine, 2-11 Inada, Obihiro 080-8555, Japan; ytakeda@obihiro.ac.jp; 2 Department of Veterinary Medicine, Obihiro University of Agriculture and Veterinary Medicine, 2-11 Inada, Obihiro 080-8555, Japan; duuya.dj@gmail.com (D.J.); chaka@obihiro.ac.jp (S.M.); hogawa@obihiro.ac.jp (H.O.); 3 Department of Chemistry, Osaka Dental University, 8-1 Kuzuha Hanazono Hirakata, Osaka 573-1121, Japan; makita@cc.osaka-dent.ac.jp; 4 Department of Oral Surgery, Ikegami General Hospital, 6-1-19 Ikegami Ootaku, Tokyo 146-8531, Japan; kaneko@ikegamihosp.jp; 5 Center of Innovation in Dental Education, Osaka Dental University, 8-1 Kuzuha Hanazono Hirakata, Osaka 573-1121, Japan

**Keywords:** disinfectants, ethanol, hand hygiene, ozonated water, SARS-CoV-2, slightly acidic electrolyzed water, virucidal activity, virucidal mechanism

## Abstract

This study aimed to compare the SARS-CoV-2-inactivation activity and virucidal mechanisms of ozonated water (OW) with those of slightly acidic electrolyzed water (SAEW) and 70% ethanol (EtOH). SARS-CoV-2-inactivation activity was evaluated in a virus solution containing 1%, 20% or 40% fetal bovine serum (FBS) with OW, SAEW or EtOH at a virus-to-test solution ratio of 1:9, 1:19 or 1:99 for a reaction time of 20 s. EtOH showed the strongest virucidal activity, followed by SAEW and OW. Even though EtOH potently inactivated the virus despite the 40% FBS concentration, virus inactivation by OW and SAEW decreased in proportion to the increase in FBS concentration. Nevertheless, OW and SAEW showed potent virucidal activity with 40% FBS at a virus-to-test solution ratio of 1:99. Real-time PCR targeting the viral genome revealed that cycle threshold values in the OW and SAEW groups were significantly higher than those in the control group, suggesting that OW and SAEW disrupted the viral genome. Western blotting analysis targeting the recombinant viral spike protein S1 subunit showed a change in the specific band into a ladder upon treatment with OW and SAEW. OW and SAEW may cause conformational changes in the S1 subunit of the SARS-CoV-2 spike protein.

## 1. Introduction

The global prevalence of severe acute respiratory syndrome coronavirus 2 (SARS-CoV-2) has not yet been fully controlled, despite the remarkable speed of development of therapeutic drugs and vaccines. To prevent further spread of infection, it is necessary to install individual infection prevention measures in daily life that can be implemented for society as a whole. SARS-CoV-2 may be transmitted through the mucous membranes of the mouth, nose and eyes through transfer onto hands, as well as via respiratory droplets. Therefore, in addition to countermeasures against the prevention of respiratory transmission, hand hygiene is also important for stricter infection control [1].

Hand hygiene involves hand washing with soap and running water. In addition, disinfection of viruses with rubbing alcohol has become mainstream worldwide [2,3]. Rubbing alcohol containing 70–80% ethanol (EtOH) can sufficiently inactivate viruses and ≥30% EtOH potently inactivated SARS-CoV-2 for 30 s when mixed with a virus solution at a virus-to-EtOH ratio of 2:8 [4]. Furthermore, a recent report showed that acidic electrolyzed water (AEW), a type of hypochlorous acid water, has an inactivating effect on SARS-CoV-2 [5,6]. Previous reports have detailed the SARS-CoV-2-inactivation activities of AEWs with different pH, namely, highly AEW (pH ~2.5) [5] and slightly AEW (SAEW; pH 5.0–5.6) [6]. SAEW with residual chlorine concentration (RCC) of ~30–50 ppm showed virucidal activity for 20 s when mixed with a virus solution at virus-to-SAEW ratios of 1:9–19 [6]. Furthermore, ozonated water (OW) with low concentrations (0.6 ppm) of ozone has been shown to reduce virus infectivity upon exposure to SARS-CoV-2 for 1 min when mixed with a virus solution at virus-to-OW ratio of 1:100 [7]. In addition, OW with a high ozone concentration (10 ppm) has been reported to inactivate SARS-CoV-2 within 5–20 s when mixed with a virus solution at virus-to-OW ratio of 1:99 [8]. Nevertheless, the SARS-CoV-2-inactivation activities of these different disinfectants have not been compared under the same experimental conditions. In this study, we evaluated the virucidal activity of OW with various ozone concentrations mixed with a SARS-CoV-2 solution at various liquid volume ratios, and this activity was compared to that of SAEW (pH 5.8) and EtOH. In addition, the impact of the organic substance content in the virus solution on these activities was evaluated. Moreover, the effects of these agents on the SARS-CoV-2 genome and S protein were analyzed to elucidate their mechanisms of action.

## 2. Materials and Methods

### 2.1. Test Solutions

OW (ozone concentration: 1–10 ppm) was generated from ultra-pure water (UPW) using a Handlex instrument (Nikka Micron Co., Ltd., Saitama, Japan). These ozone concentrations were determined based on the standard ozone concentrations of OW provided by an OW generator. The range of tested concentration of ozone (1–10 ppm) included low (1 ppm) and high (10 ppm) concentrations that are close to or consistent with previously reported virucidal concentrations [7,8]. The ozone concentration was measured using an OZ-20 ozone meter (DDK-TOA Co., Tokyo, Japan). SAEW (pH ~5.8, RCC ~34 ppm, oxidation-reduction potential ~950 mV) was generated by the electrolysis of hydrochloric acid using a PURESTER device (Morinaga Milk Industry Co., Ltd., Tokyo, Japan). The tested RCC was determined based on the recommended concentration for SARS-CoV-2 inactivation in Japan [9], which is empirically considered to have a low risk of skin irritation. The pH, RCC and oxidation-reduction potential were measured using a compact pH meter (Horiba Co., Ltd., Kyoto, Japan), AQUAB AQ-202 (Sibata Scientific Technology Ltd., Tokyo, Japan) and a waterproof oxidation-reduction potential meter (Custom Co., Tokyo, Japan), respectively. EtOH (70%) was prepared by diluting 100% EtOH (Fujifilm Wako Pure Chemical Co., Ltd., Osaka, Japan) with UPW.

### 2.2. Virus and Cells

SARS-CoV-2 (JPN/TY/WK-521 strain) was obtained from the National Institute of Infectious Diseases (Tokyo, Japan). VeroE6/TMPRSS2 cells [10] were obtained from the Japanese Collection of Research Bioresources (Osaka, Japan, Cell No. JCRB1819). SARS-CoV-2-infected VeroE6/TMPRSS2 cells were cultured in virus growth medium (VGM), the composition of which has been previously described [11].

### 2.3. Evaluation of the Virucidal Activity of Test Solutions

The VGM containing SARS-CoV-2 with 1–40% (*v*/*v*) fetal bovine serum (FBS), in which the viral titer was ~7.0 log_10_ 50% tissue culture infective dose (TCID_50_)/mL, was mixed with the test solutions at virus-to-test solution ratios of 1:9, 1:19 and 1:99. As a control, the virus solution was mixed with the UPW. The mixtures were incubated for 20 s at 22 °C and then inoculated into the cells cultured in VGM containing 10 mM Na_2_S_2_O_3_, which is an ozone and chlorine neutralizer. Ten-fold serial dilutions of the cell culture medium were performed. After incubation for 3 days, the viral titers (TCID_50_/mL) were evaluated as previously described [11]. EtOH carried into the cell culture medium did not affect virus proliferation in infected cells and did not inactivate the viruses. The virus-to-test solution ratios and reaction times were set to broadly cover the experimental conditions used in related previous studies [4,6,7,8]. The tested FBS concentrations were determined based on our previous study, in which the SARS-CoV-2-inactivation activity of AEW was evaluated [5]. The detection limit of the viral titer in each test solution group was determined based on the cytotoxicity of each test solution. UPW, SAEW and OW did not show any cytotoxicity, and the detection limit of the viral titer in the groups treated with these solutions was set to 1.25 log_10_ TCID_50_/mL according to our viral titer calculation. In contrast, 70% EtOH exhibited a moderate cytotoxicity. The detection limit in the 70% EtOH group was set to 2.25 log_10_ TCID_50_/mL.

### 2.4. Real-Time Reverse Transcription-Polymerase Chain Reaction (RT-PCR) Analysis

The following two treatments were performed: In the first treatment (pre-addition of Na_2_S_2_O_3_), Na_2_S_2_O_3_ was added to the test solutions (34 ppm SAEW, 10 ppm OW and 70% EtOH), with a final Na_2_S_2_O_3_ concentration of 10 mM. According to this treatment, the chlorine in the SAEW and ozone in the OW were neutralized. The VGM containing SARS-CoV-2 with 1% FBS was then mixed with the test solutions at a virus-to-test solution ratio of 1:29. The mixture was incubated for 20 s at 22 °C. In the second treatment (post-addition of Na_2_S_2_O_3_), the VGM containing SARS-CoV-2 with 1% FBS was mixed with the test solutions at a virus-to-test solution ratio of 1:29. The mixture was incubated for 20 s at 22 °C. Na_2_S_2_O_3_ was then added to the mixture.

RNA was extracted from these mixtures obtained by pre- and post-treatment with Na_2_S_2_O_3_, and real-time RT-PCR targeting the SARS-CoV-2 nucleocapsid (*N*) gene was performed as previously described [11]. The viral titers of these mixtures were also evaluated.

### 2.5. Western Blotting (WB) Analysis

The following two treatments were performed using recombinant SARS-CoV-2 S protein S1 subunit (Catalog No. 40591-V08H; Sino Biological Inc., Beijing, China) and test solutions.

In the first treatment (pre-addition of Na_2_S_2_O_3_), Na_2_S_2_O_3_ was added to each test solution. The recombinant protein solution (250 μg/mL) was mixed with the test solutions at a protein-to-test solution ratio of 1:29. The mixture was incubated for 20 s at 22 °C. In the second treatment (post-addition of Na_2_S_2_O_3_), the recombinant protein solution was mixed with the test solutions at a protein-to-test solution ratio of 1:29. The mixture was incubated for 20 s at 22 °C, after which Na_2_S_2_O_3_ was added to the mixture. 

The mixtures obtained by the two treatments were combined with sodium dodecyl sulfate (SDS) buffer containing 2-mercaptoethanol (FUJIFILM Wako Pure Chemical Co., Ltd.). These SDS samples were subjected to WB analysis targeting the S protein S1 subunit, as previously described [11].

### 2.6. Statistical Analysis

Student’s *t*-test was performed to analyze the statistical significance of the differences in the viral titer between the UPW (control) group and each test solution group. Statistical significance was set at *p* < 0.05.

## 3. Results

### 3.1. Comparison of the Virucidal Activities of SAEW, OW and 70% EtOH

**Figure 1:**
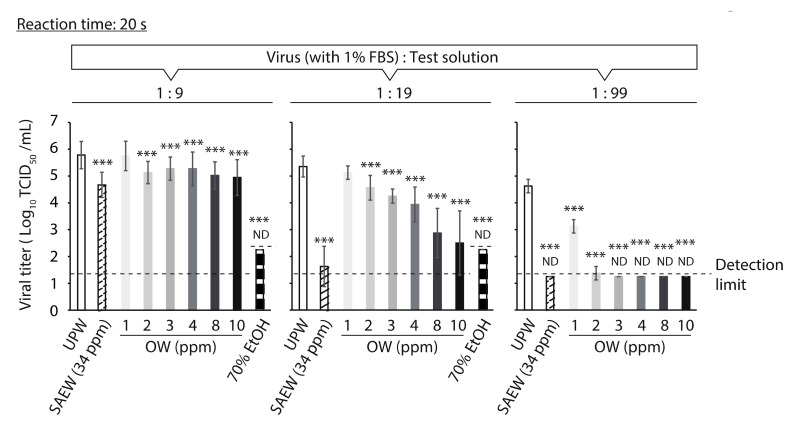
Comparison of virucidal activities of slightly acidic electrolyzed water (SAEW), ozonated water (OW) and 70% ethanol (EtOH). The viral titer of each reaction mixture was measured. Results are indicated as mean ± standard deviation (*n* = 3–30 per group). The detection limits of the viral titer are 1.25 log_10_ 50% tissue culture infective dose (TCID_50_)/mL in the ultra-pure water (UPW), SAEW and OW groups, and 2.25 log_10_ TCID_50_/mL in the 70% EtOH group. ND is the abbreviation for not detected, which means that the viral titer was below the detection limit. Student’s *t*-test was performed to analyze the statistical significance between the UPW (control) group and each test solution group; *** *p* < 0.001. FBS, fetal bovine serum.

The SARS-CoV-2-inactivation activities of SAEW, OW and 70% EtOH were compared. When the virus solution (1% FBS concentration) and test solutions were mixed at a virus-to-test solution ratio of 1:9, the viral titer of the 70% EtOH group was below the detection limit (≥3.53 log_10_ TCID_50_/mL reduction of viral titer compared to that in the UPW group) after 20 s. In contrast, 34 ppm SAEW and 2–10 ppm OW showed weak and limited virucidal activity (Figure 1, left). At a ratio of 1:19, SAEW and 2–10 ppm OW showed more potent virucidal activities (SAEW: ≥ 3.73 log_10_ TCID_50_/mL reduction; 2 ppm OW: 0.79 log_10_ TCID_50_/mL reduction; 10 ppm OW: ≥ 2.85 log_10_ TCID_50_/mL reduction) than those at a ratio of 1:9 after 20 s (Figure 1, middle). At a ratio of 1:99, the viral titers in the SAEW and 2–10 ppm OW groups were almost below the detection limit after 20 s (≥3.25 log_10_ TCID_50_/mL reduction) (Figure 1, right).

### 3.2. Evaluation of the Impact of FBS in Virus Solution on the Virucidal Activity of Test Solutions

**Figure 2:**
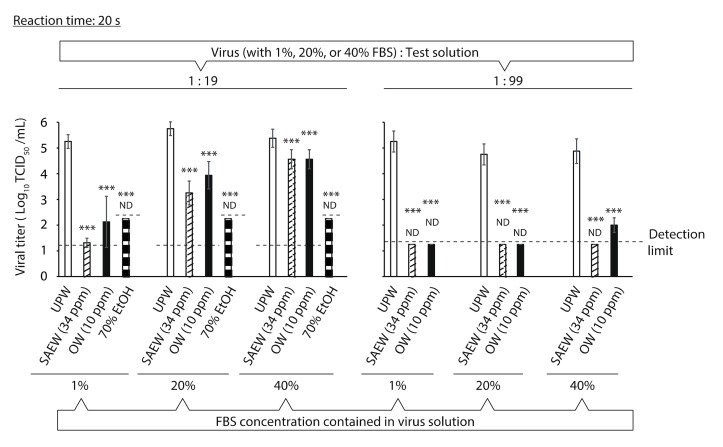
Evaluation of the impact of fetal bovine serum (FBS) concentration in the virus solution on the virucidal activities of test solutions. The viral titer of each reaction mixture was measured. Results are indicated as mean ± standard deviation (*n* = 4–8 per group). The detection limits of the viral titer are 1.25 log_10_ 50% tissue culture infective dose (TCID_50_)/mL in the ultra-pure water (UPW), slightly acidic electrolyzed water (SAEW) and ozonated water (OW) groups, and 2.25 log_10_ TCID_50_/mL in the 70% ethanol (EtOH) group. ND is the abbreviation for not detected, which means that the viral titer was below the detection limit. Student’s *t*-test was performed to analyze the statistical significance between the UPW (control) group and each test solution group; *** *p* < 0.001.

The virucidal activities of the test solutions were evaluated against virus solutions with different FBS concentrations (1%, 20% and 40%). At a virus-to-test solution ratio of 1:19, the viral titer in the 70% EtOH group was below the detection limit after 20 s even though the FBS concentration in the virus solution was 40% (≥3.13 log_10_ TCID_50_/mL reduction compared to that in the UPW group). In contrast, the reduction in viral titer by 34 ppm SAEW and 10 ppm OW decreased in proportion to the increase in FBS concentration of the virus solution (Figure 2, left). At a ratio of 1:99, the viral titer in the SAEW group was below the detection limit after 20 s despite the 40% FBS setting (≥3.63 log_10_ TCID_50_/mL reduction). In contrast, the viral titer in the 10 ppm OW group was not below the detection limit in the 40% FBS setting. Nevertheless, the reduction in viral titer with OW treatment was ≥2.88 log_10_ TCID_50_/mL (Figure 2, right).

### 3.3. Evaluation of the Impact of Test Solutions on the SARS-CoV-2 Genome

**Figure 3:**
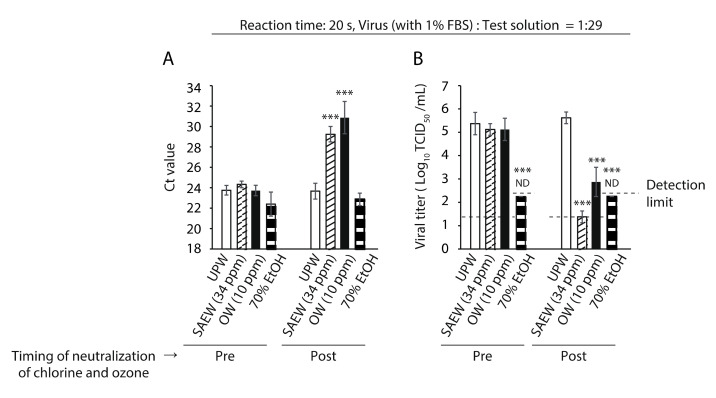
Evaluation of the impact of test solutions on the SARS-CoV-2 genome. (**A**) The cycle threshold (Ct) value of each reaction mixture was measured. (**B**) The viral titer of each reaction mixture was measured. The detection limits of the viral titer are 1.25 log_10_ 50% tissue culture infective dose (TCID_50_)/mL in the ultra-pure water (UPW), slightly acidic electrolyzed water (SAEW) and ozonated water (OW) groups, and 2.25 log_10_ TCID_50_/mL in the 70% ethanol (EtOH) group. ND is the abbreviation for not detected, which means that the viral titer was below the detection limit. (A, B) Results are indicated as mean ± standard deviation (*n* = 4 per group). Student’s *t*-test was performed to analyze the statistical significance between the UPW (control) group and each test solution group; *** *p* < 0.001. Pre indicates that Na_2_S_2_O_3_ was added to each test solution before mixing with the virus solution; Post indicates that Na_2_S_2_O_3_ was added 20 s after mixing with the virus solution. FBS, fetal bovine serum.

Real-time RT-PCR targeting the viral genome showed that the cycle threshold (Ct) values in all tested groups were comparable in the case of Na_2_S_2_O_3_ pre-addition, whereas these values in the SAEW and OW groups were significantly higher than those in the UPW group in the case of Na_2_S_2_O_3_ post-addition. However, the Ct value in the 70% EtOH group was comparable to that in the UPW group (Figure 3A). In this setting, the virucidal activities of SAEW and OW disappeared with pre-addition of Na_2_S_2_O_3_, indicating that the chlorine in SAEW and the ozone in OW were neutralized by the addition of Na_2_S_2_O_3_ (Figure 3B).

### 3.4. Impact of Test Solutions on the SARS-CoV-2 S Protein

**Figure 4:**
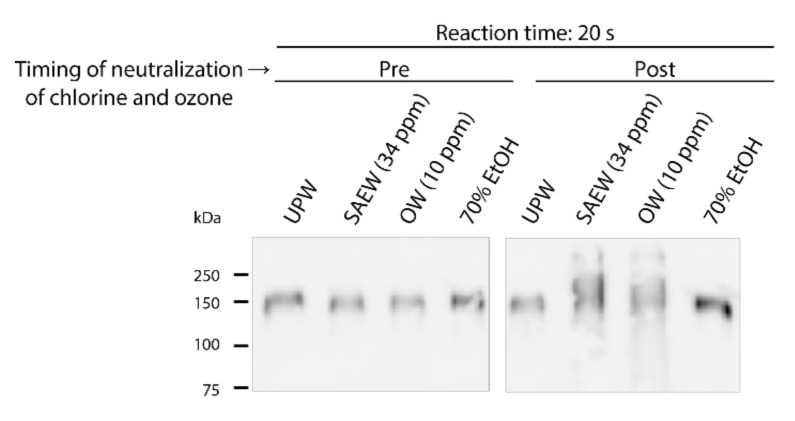
Impact of test solutions on the SARS-CoV-2 S protein. Images of the results of Western blotting analysis targeting the SARS-CoV-2 S protein S1 subunit are shown. Pre indicates that Na_2_S_2_O_3_ was added to each test solution before mixing with the recombinant S protein S1 subunit; Post indicates that Na_2_S_2_O_3_ was added 20 s after mixing with the S1 subunit. UPW, ultra-pure water; SAEW, slightly acidic electrolyzed water; OW, ozonated water; EtOH, ethanol.

WB analysis showed no differences in the band patterns of the recombinant S protein S1 subunit among the proteins after treatment with all test solutions in the case of Na_2_S_2_O_3_ pre-addition. The band pattern of the protein treated with 70% EtOH was comparable to that of the protein treated with UPW in the case of Na_2_S_2_O_3_ post-addition. However, the band changed into a ladder for proteins following treatment with SAEW and OW in the case of Na_2_S_2_O_3_ post-addition (Figure 4).

## 4. Discussion

OW with a high concentration (10 ppm) of ozone has been reported to be effective in rapidly inactivating SARS-CoV-2 at a virus-to-test solution ratio of 1:99 [8]. However, the inactivation activity of OW against SARS-CoV-2 has not been fully evaluated. For example, the activity of disinfectants is greatly reduced by the influence of organic substances, and the effects of disinfectants largely differ depending on the test conditions [12]. Therefore, in this study, OW with various ozone concentrations and liquid volume ratios was mixed with virus solutions containing various concentrations of FBS, and its virucidal activity was compared with that of SAEW and 70% EtOH. The results showed that the virus inactivation activity of 70% EtOH was the strongest in comparison with that of 10 ppm OW and SAEW (RCC: ~34 ppm). In addition, the inactivation activity of SAEW (~34 ppm) was slightly stronger than that of OW (10 ppm). When 10 μL of the VGM containing ~5.0 log_10_ TCID_50_ SARS-CoV-2 and 40% FBS was reacted with 990 μL of 10 ppm OW (virus-to-test solution ratio of 1:99), SARS-CoV-2 could be sufficiently inactivated (specifically, ≥2.88 log_10_ TCID_50_/mL reduction of the viral titer; ≥99.87% inactivation of SARS-CoV-2) after 20 s, even in the presence of a high concentration of organic substances (Figure 2, right). The viral titer (~5.0 log_10_ TCID_50_) of the tested virus solution was presumed to be higher than that of the virus on a COVID-19 patient’s hand in the real world; Lin et al. [13] reported that the viral titer of SARS-CoV-2 detected on a COVID-19 patient’s hand was up to ~3 × 10^3^ plaque-forming unit/hand. Nevertheless, the current study has some limitations. First, our results cannot be fully translated to the efficacy of OW and SAEW in the real world because the compositions of substances contained in virus solutions are different. In addition, the influence of the substances contained in VGM on the virucidal activity of each test solution was not clarified in the current study. To assess the direct virucidal activity of each test solution, an additional analysis should be performed targeting purified SARS-CoV-2. Nevertheless, this in vitro study suggests that OW and SAEW may be useful SARS-CoV-2 disinfectants in situations such as hand disinfection under running water.

One of the disadvantages of OW is the short half-life of ozone after OW generation. The half-life of ozone in distilled water is reported to be 20 to 30 min at 20 °C; it is also reported to be 2–4 min in a pH 7.0 aqueous solution at 25 °C [14]. Owing to the short half-life of ozone, OW should be used for hand or object washing with running water. In contrast, our previous study showed that the RCC of SAEW does not dramatically decrease for 21 days in an enclosed container at 22 °C in the dark [6]. Nevertheless, AEW users need to consider that the available chlorine in AEW is decomposed by ultraviolet light, and this reduction speed is accelerated at high temperatures. Conversely, although EtOH is also lost by evaporation, it can be maintained for a long time under enclosed conditions. Even though EtOH is more stable than OW and SAEW, its skin irritation and the existence of alcohol-intolerant people are disadvantages. Previous reports suggest that the skin irritancy of OW is lower than that of EtOH [15,16]. In contrast, since SAEW is also considered to be safe and has low irritation to skin and mucous membranes, it has been proposed for use as a disinfectant for normal and wounded skin [17]. In addition to the low irritancy of OW and SAEW, they are less of a pollution risk for the environment, making them advantageous as disinfectants. Hence, OW and SAEW can be used as surrogate disinfectants of EtOH, especially for people who show adverse reactions to alcohol.

Previous studies have shown that ozone [18] and hypochlorous acid [19] damage viral proteins, genomes and lipid layers of biological membranes, such as the viral envelope. Alcohols show more substantial virucidal activity against enveloped viruses than non-enveloped viruses, suggesting that the viral lipid envelope is a potential target of EtOH [20]. However, there are no reports of comparative studies of these disinfectants for the inactivation of SARS-CoV-2 at the molecular level. In this study, real-time RT-PCR targeting the viral genome suggested that OW and SAEW may have destroyed the viral genome in a short time. In contrast, 70% EtOH had no effect on the viral genome at a reaction time of 20 s. This finding for EtOH is consistent with a report by Pfaender et al. [21], which showed that EtOH does not affect the integrity of the viral RNA of the hepatitis C virus. In general, when performing PCR screening of samples from patients suspected to be infected with SARS-CoV-2, it is important to prevent contamination of the viral genome between samples. Our real-time RT-PCR results suggest that OW and SAEW, which can rapidly inactivate SARS-CoV-2 with destruction of the viral genome, may be useful virucidal disinfectants in such situations. 

The results of the WB analysis further demonstrated that OW and SAEW may have caused some conformational changes in the S1 subunit of the S protein within 20 s. Rowen et al. [22] stated that ozone can directly inactivate many viruses. They mentioned the possibility that ozone can oxidize the glycoprotein on viral particles, transforming it from the reduced form (R-S-H) to the oxidized form (R-S-S-R). We were not able to evaluate the impact of the test agents on the viral envelope in this study. Nevertheless, considering previous studies suggesting the impacts of ozone, hypochlorous acid and alcohol on biological membranes, the test agents might have affected the SARS-CoV-2 envelope, which also contributed to virus inactivation.

In conclusion, sufficient amounts of OW (10 ppm) and SAEW (34 ppm) could achieve effective SARS-CoV-2 inactivation rapidly, even in the presence of high concentrations of organic substances. Our results further revealed that OW and SAEW affected the genome and S protein of SARS-CoV-2; however, no such effect was observed with EtOH treatment under the same reaction conditions. Even though further clinical studies are needed in the future, we believe that both EtOH and OW or SAEW may be effective as SARS-CoV-2 disinfectants for actual hand hygiene, depending on the cleaning method, time and concentration.

## Data Availability

Data is not available from the authors.

## Sample Availability

Samples of the compounds are not available from the authors.
